# High-Efficiency Ultrathin Si-Based Solar Cells by Cascading Dilute-Nitride GaNAsP

**DOI:** 10.3390/ma14237415

**Published:** 2021-12-03

**Authors:** Yen-Ju Lin, David Jui-Yang Feng, Tzy-Rong Lin

**Affiliations:** 1 Institute of Applied Mechanics, National Taiwan University, Taipei 106319, Taiwan; f98543039@ntu.edu.tw; 2 Department of Electrical Engineering, National University of Kaohsiung, Kaohsiung 811726, Taiwan; 3 Institute of Optoelectronic Sciences, National Taiwan Ocean University, Keelung 20224, Taiwan; 4 Department of Mechanical and Mechatronic Engineering, National Taiwan Ocean University, Keelung 20224, Taiwan; 5 Center of Excellence for Ocean Engineering, National Taiwan Ocean University, Keelung 20224, Taiwan

**Keywords:** dilute-nitride, GaNAsP, ultrathin solar cell, Si-based

## Abstract

Thin-film solar cells are currently an important research subject. In this study, a lattice-matched GaNAsP/Si tandem cell was designed. We adopted the drift-diffusion model to analyze the power conversion efficiency (PCE) of the solar cell. To find the maximum solar cell PCE, the recombination terms and the interlayer between subcells was omitted. For an optimal tandem cell PCE, this study analyzed the mole fraction combinations of GaNAsP and the thickness combinations between the GaNAsP and the Si subcells of the tandem cell. Our results showed the superiority of the tandem cell over the Si cell. The 4.5 μm tandem cell had a 12.5% PCE, the same as that of the 10.7 μm Si cell. The 11.5 μm tandem cell had 20.2% PCE, while the 11.5 μm Si cell processed 12.7% PCE. We also analyzed the Si subcell thickness ratio of sub-12 μm tandem cells for maximum PCE. The tandem cell with a thickness between 40% to 70% of a Si cell would have a max PCE. The ratio depended on the tandem cell thickness. We conclude that the lattice-matched GaNAsP/Si tandem cell has potential for ultrathin thin Si-based solar cell applications.

## 1. Introduction

Photovoltaic (PV) technology is a renewable and environment-friendly power source that can help satisfy the growing energy demand. Crystalline silicon (c-Si)-based solar cells are commonly used to generate solar power owing to their widespread availability. Owing to their indirect bandgap, Si solar cells should have sufficient thickness to overcome their weaker absorption strength [1]. Petermann et al. proposed a crystal silicon solar cell with 19% power conversion efficiency (PCE) containing 43 μm thick Si [2]. With the increase in demand for low-cost, flexible, and lightweight electric devices, the demand for thin-film solar cells has grown in recent years. Thin-film c-Si solar cells are bendable and flexible, and they have been extensively studied [3]. A Si-based ultra-thin solar cell with a thickness below 10 μm has been reported, but unfortunately, its PCE is limited to less than 10% [4,5].

The low PCE of ultrathin Si-based solar cells is primarily due to insufficient absorption in the cell. The following well-known methods are typically adopted to enhance the absorption of PV devices, including: (1) reducing the reflection on the front surface to increase the number of photons entering the cell; (2) increasing the travel path of the photons inside the cell; and (3) cascading several solar cells, with each cell having different energy bandgap. The first two methods are called light trapping methods. Recent investigation into surface texturing, including nanostructure growing [6,7,8,9], and metal-assisted etching [10], have demonstrated good anti-reflection properties. Some studies have shown that putting metal nanoparticles on the Si surface or inside the Si can increase the absorption strength through the plasmonic effect [11,12]. These methods can be used to improve the absorption coefficient strength to enhance photon-generated current, however, the difficulty of nanostructure formation on the Si surface increases with the reduction in thickness.

Utilizing different bandgaps to expand the absorbed spectrum range and form a tandem cell has been utilized to improve the PCE. A perovskite/Si tandem cell can achieve a PCE of approximately 25% with about 250 μm thick Si [13,14], however, it is not bendable. To prevent the PCE loss of Si-based tandem cells from defects during the epitaxy process, a Si-lattice-matched material is required to reduce the number of defects. GaNAsP is a good candidate material. It has a large lattice constant modulation and can have the same lattice constant as Si. Nacer et al. demonstrated the possibility of a lattice-matching GaNAsP/Si solar cell [15]. Their simulation determined the thickness and concentration of GaNAsP from current-matched conditions. Their optimal design achieved 37.5% PCE with a Si bottom cell and sufficient thickness for complete absorption. Zhang et al. proposed a lattice-matched monolithic GaNP/GaNAsP/Si triple-junction solar cell. By adjusting the GaNP cell and GaNAsP cell thicknesses, the triple-junction solar cell achieved a maximum PCE of 41.53% with low surface recombination velocity and long carrier lifetime [16]. However, the Si bottom cell had a thickness of 300 μm, too thick to utilize in a thin-film solar cell. Sertel et al. ultized molecular beam epitaxy (MBE) to grow GaPAsN on a Si and a GaP substrate with N concentration, N(*x*), of 0.462% and 0.409%, respectively [17]. The GaNAsP epilayer exhibited high crystal quality. Yu et al. grew 0.5 μm GaNAsP on a GaAs substrate by metal organic chemical vapor deposition (MOCVD), which N concentrations were reported in the range of 0.3–1% [18]. Zelazna et al. grew 100 nm GaNAsP with 2.5% N on a GaP substrate by MBE [19].

An intermediate band solar cell (IBSC) can improve the PCE of a solar cell [20]. By inserting an intermediate band (IB) in the energy gap, the solar cell can use low-energy photons to increase the photon-generated current, with an open voltage similar to the band-gap energy. GaNAsP can be applied for IBSC because its conduction band (CB) splits into two bands ($E_{c}^{+}$ with higher energy and $E_{c}^{-}$ with lower energy) due to the band anti-crossing effect, wherein the $E_{c}^{-}$ band can be used as an IB [21]. Jussila et al. demonstrated the importance of the interlayer and the doping concentration on GaNAsP-based IBSC [22]. They compared the IBSC performance under the extreme condition of IB’s doping concentration and recombination intensity. This result gave the upper and lower limit of PCE of GaNAsP-based IBSC. However, their studies only considered the energy gap segmentation of the IB without considering the difference in the absorption strength of the two sections with regard to the IB. The absorption spectra of the two sections are assumed to have the same strength, which is favorable for the ideal IBSC model. However, we believe that it over-estimates the PCE of the GaNAsP-based IBSC.

In this work, for achieving which Si-based devices can be more bended, flexible and transparent as reported in [3], we aim to present a GaNAsP/Si tandem cell with a thickness <15 mm. The absorption spectrum of GaNAsP was calculated instead of assuming three-section absorption spectrum with the same strength to analyze GaNAsP-based IBSC with $E_{c}^{-}$ as the IB. GaNAsP with the same lattice constant as Si was also analyzed. A GaNAsP/Si tandem cell was designed from the lattice-matched characteristic. Finally, the PCEs of the tandem cells were calculated with GaNAsP concentration modulation. The PCEs of GaNAsP/Si tandem cells consisting of different thickness combinations of GaNAsP cell and Si cells, were also analyzed.

## 2. Theory

The band structure of GaN*_x_*AsP*_y_* can be calculated using the band anti-crossing (BAC) model [21,23]. The BAC model describes how the band structure of CB is affected by adding nitrogen into GaAsP*_y_*. The BAC model is presented in Equations (1) and (2):

(1) $$H_{BAC}=\left(\begin{array}{cc} E_{M}\left(k\right) & C_{NM}\sqrt{x}\\ C_{NM}\sqrt{x} & E_{N} \end{array}\right)$$

(2) $$C_{NM}=\frac{C_{N\Gamma }}{{\left[1+{\left(ak\right)}^{2}\right]}^{2}}$$

where *E*_*M*_ (*k*) represents the band edge of GaAsP*_y_*, *E*_N_ represents the energy of nitrogen level above the valence band of GaAsP*_y_*, *C*_N*M*_ is the coupling constant between nitrogen level and the CB, *C*_NT_ is the coupling constant at the Γ point, *a* is the distance between the *k* point and the Γ point, and *x* is the concentration of nitrogen. The material parameters (taken from [24]) are: *E*_N_ = 1.65 eV and $C_{NM}$ = 2.8 eV for GaNAs [21,23], and *E*_N_ = 2.18 eV and *C*_N*M*_ = 3.05 eV for GaNP [25] are utilized. The CB of GaNAsP splits into two bands due to the band anti-crossing effect; the high energy band is

$$E_{c}^{+}$$

band, and the lower energy band is $E_{c}^{-}$ band. The $E_{c}^{+}$ band and $E_{c}^{-}$ band can be calculated using the band anti-crossing model as shown in Equation (3):

(3) $$E_{c}^{\pm }\left(k\right)=\frac{E_{M}\left(k\right)+E_{N}\pm \sqrt{{\left(E_{M}\left(k\right)-E_{N}\right)}^{2}+4xC_{NM}^{2}}}{2}$$

The $E_{c}^{-}$ band can be regarded as the IB, and the $E_{c}^{+}$ band can be regarded as the CB. The valence band (*E*_v_) is the same as that of GaAsP. In addition, to prevent carrier outflow through the IB, GaP is used as the material for the P- and N- regions. The optical properties should be estimated first, especially the ability of photon absorption for PCE analysis of solar cells. The absorption spectrum, α(ℏω), can be calculated using Equations (4) and (5):

(4) $$\alpha \left(\hbar \omega \right)=C_{0}{\left|e\cdot p\right|}^{2}\rho_{r}\left(\hbar \omega -E_{g}\right)$$

(5) $$C_{0}=\frac{\pi e^{2}}{n_{r}c\varepsilon_{0}m_{0}^{2}\omega }$$

where *ρ_r_* is the joint density of states, ${\left|e\cdot p\right|}^{2}$ is the optical matrix element, *e* is the electron charge, *c* is the speed of light in vacuum, *m*_0_ is the electron mass, *n_r_* is the refractive index, *ε*_0_ is the vacuum permittivity [26], and *ω* is the light frequency. The joint density of states must be derived to analyze the optical properties of bulk GaNAsP because the $E_{c}^{-}$ and $E_{c}^{+}$ bands are not calculated from the effective mass model with parabolic approximation. The joint density of states can be derived from the transition energy, using Equation (3), and the dispersion relation of the VB from the one-band effective mass method. Further, some modifications must be made for the calculation of the momentum matrix elements. According to the BAC model, electron wave functions consist of basis functions with an *S* orbit function and *N* level. Duboz [27] revealed that the *N* level base acts as a defect level that cannot contribute to optical transition; only the *S* orbit component can participate. Consequently, the optical matrix must be modified by a factor, as shown in Equation (6):

(6) $$\begin{array}{ll} d & =\;\left(a_{1}\langle \Psi_{\Gamma 1}|+b_{1}\langle \Psi_{N}|\right)z\left(a_{n}|\Psi_{\Gamma n}\rangle +b_{n}|\Psi_{N}\rangle \right)\\ & =\;a_{1}^{*}a_{n}\langle \Psi_{\Gamma 1}|z|\Psi_{\Gamma n}\rangle \end{array}$$

GaNAsP can be applied in an IBSC [21]. The method and the structure proposed by Yoshida et al. [28] is employed herein to analyze the PCE of an IBSC. The cell considered in [28] had a 0.5 μm thick P-region, 1 μm thick I-region and 2 μm thick N-region. The doping concentrations of P- and N- regions were 7 × 10^17^ cm^−3^ and 6 × 10^17^ cm^−3^, respectively. In this study, the I-region material is pre-doped to ensure that the IBSC meets the carrier balance condition in the IB. The doping concentration of I-region material depends on the strength of two section absorption spectrum: the transition from the valence band to the intermediate band, *α* (VB, IB), and the transition from the intermediate band to the conduction band, *α* (IB, CB). The recombination terms were ignored to simplify the calculation.

In this work, we tried to design a lattice-matched GaNAsP/Si tandem cell. First, the concentration combination was analyzed to be lattice-matched with GaP and Si. Then the absorption spectrum was calculated for solar cell power conversion efficiency (PCE) analysis. Finally, thickness combinations of GaNAsP solar cell and Si solar cell were calculated to find the combination of thickness with the maximum PCE of lattice-matched GaNAsP/Si tandem cell.

## 3. Numerical Results and Discussion

### 3.1. GaNAsP Lattice Match with GaP

In this section, we analyze the concentration of GaNAsP required to have the same lattice constant as that of GaP. The lattice constants (*a*_0_) of III-V compounds and the interpolation equation of the GaNAsP lattice constant are the same as those obtained by Vurgaftman et al. in [24]:

(7) $$a_{0}:\;{\mathrm{GaN}}_{x}{\mathrm{AsP}}_{y}=\frac{xy{\mathrm{GaN}}_{u}P+y\left(1-x-y\right){\mathrm{GaAs}}_{v}P+x\left(1-x-y\right){\mathrm{GaNAs}}_{w}}{xy+y\left(1-x-y\right)+x\left(1-x-y\right)}$$

(8) $$u\equiv \frac{1+x-y}{2},\;v\equiv \frac{2-x-2y}{2},\;w\equiv \frac{2-2x-y}{2}$$

(9) $$\mathrm{Ga}N_{u}P=u\mathrm{GaN}+\left(1-u\right)\mathrm{GaP}$$

(10) $$\mathrm{GaA}s_{v}P=v\mathrm{GaAs}+\left(1-v\right)\mathrm{GaP}$$

(11) $$\mathrm{GaNA}s_{w}=\left(1-w\right)\mathrm{GaN}+w\mathrm{GaAs}$$

**Figure 1:**
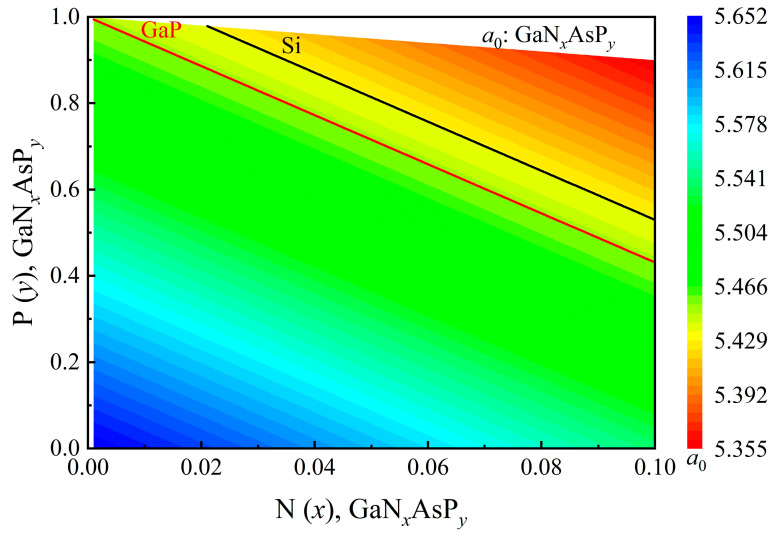
Lattice constant (*a*_0_) variation for GaN*_x_*As_1-*x*-*y*_P*_y_* with different mole fractions of N(*x*) and P(*y*). Redline represents mole fraction combinations to be lattice-matched with Si; black line represents the combinations to be lattice-matched with GaP.

The lattice constant of GaNAsP is calculated by an interpolation formula of related ternary materials: GaNP, GaAsP, and GaNAs. Vegard’s law obtains the lattice constant of ternary material with binary compounds, as depicted in Equations (9)–(11). The lattice constant of binary compound is 4.5 Å for GaN, 5.65325 Å for GaAs, and 5.4505 Å for GaP [24]. The analyzed range of nitrogen (N) mole fraction is from 0.001 to 0.1. The lattice constant of GaNAsP as a function of mole fraction combination of N and phosphorus (P) is plotted in Figure 1. The summation of mole fractions of V elements must not be over 1. Therefore, there is a blank region in the lattice-constant distribution diagram. Using Equation (7), we can obtain the mole fraction combinations of GaP-lattice-matched GaNAsP and Si-lattice-matched GaNAsP. The relation between N mole fraction and P mole fraction for GaP-lattice-matched GaNAsP and Si-lattice-matched GaNAsP are shown in Equation (12). For GaP-lattice-matched GaN*_x_*AsP*_y_* and for Si-lattice-matched GaN*_x_*AsP*_y_* (the lattice constant of Si is 5.431 Å):

(12) $$\{\begin{array}{l} a_{0}^{\mathrm{GaNAsP}}=a_{0}^{\mathrm{GaP}},\;a_{0}^{\mathrm{Si}}\\ x>0,\;y>0,x+y<1\; \end{array}$$

**Figure 2:**
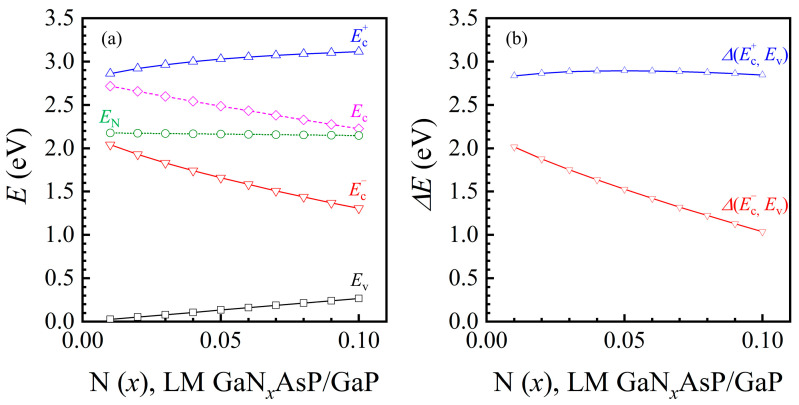
(**a**) Calculated $E_{c}^{+}$ and $E_{c}^{-}$ band edges of GaP-lattice-matched GaN*_x_*AsP*_y_*, as a function of N mole fraction. The reference energy level is the valence band edge of GaP. The valence band edge ($E_{v}$), the conduction band edge ($E_{c}$), and the nitrogen level ($E_{N}$) of the related GaAsP*_y_*; (**b**) Calculated GaP-lattice-matched GaN*_x_*AsP (LM GaN*_x_*AsP with GaP) energy gap (Δ*E*) between $E_{c}^{+}$ and valence band ($E_{v}$), Δ*E* ($E_{c}^{+}$, *E*_v_), and between $E_{c}^{-}$ and $E_{v}$, Δ*E* ($E_{c}^{-}$, $E_{v}$), as a function of N mole fraction.

The black line and red line in Figure 1 indicate the mole fraction combinations of GaNAsP with lattice-matched with Si or GaP, respectively. The energy band edges of GaP-lattice-matched GaN*_x_*AsP*_y_* calculated using the BAC-model are plotted in Figure 2a. The valence band edges (*E*_v_), conduction band edges ($E_{c}$), and the nitrogen levels (*E*_N_) of GaAsP*_y_* are also shown in the figure. The reference energy level is the valence band edge of GaP. The mole fraction combination of GaN*_x_*AsP*_y_* is depicted as the red line in Figure 1. From Figure 1, the P mole fraction in the GaP-lattice-matched mole fraction combination decreases as the N mole fraction increases. Therefore, the *E*_c_ of GaAsP*_y_* decreases, and the $E_{v}$ of GaAsP*_y_* increases with a decrease in phosphorus concentration. By contrast, the decrease in *E*_N_ is 30 meV with N mole fraction varying from 0.01 to 0.1. As the value of *E*_c_ and *E*_N_ get closer, and the energy gap between $E_{c}^{+}$ and $E_{c}^{-}$ increases with the increase in N mole fraction. The bandgap between the $E_{c}^{-}$ band and the valence band, Δ*E* ($E_{c}^{-}$, *E*_v_), decreases with increasing N concentration. However, the bandgap between the $E_{c}^{+}$ band and *E*_v_, Δ*E* ($E_{c}^{+}$, *E*_v_), increases with the increase in N concentration until N(*x*)= 0.05, it then starts to decay with the increase in N concentration, as illustrated in Figure 2b. The energy difference of Δ*E* ($E_{c}^{-}$, *E*_v_) between N(*x*)= 0.1 and N(*x*)= 0.01 is 977 meV, whereas the energy difference in Δ*E* ($E_{c}^{+}$, *E*_v_) is 61 meV. From this variance, we can conclude that the effect of N on the bandgap of GaNAsP is larger than the bandgap narrowing effect of decreasing P concentration for N concentration below 0.05. After N concentration exceeds 0.05, the bandgap narrowing effect becomes stronger.

**Figure 3:**
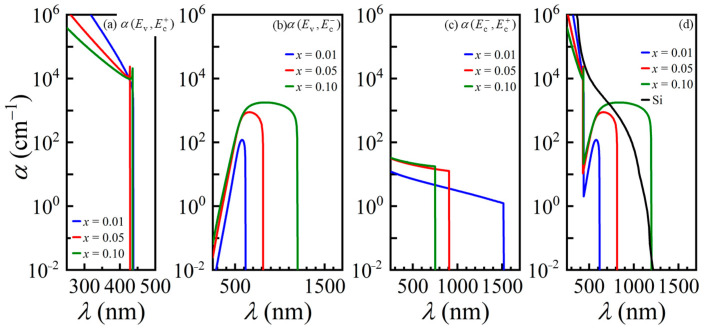
Absorption spectra (*α*) for bulk GaP-lattice-matched GaN*_x_*AsP. (**a**–**c**) Three-section absorption spectra for N = 0.01 to N = 0.1 with $E_{c}^{-}$ as an IB; indexes represent the initial and final bands of the absorption process: valence ($E_{v}$), $E_{c}^{+}$, and $E_{c}^{-}$ bands; (**d**) Absorption spectrum of GaNAsP as a single-band-gap material with different mole fractions of N. Absorption spectrum of a single-band-gap GaNAsP is the sum of *α* ($E_{v}$, $E_{c}^{+}$) and *α* ($E_{v}$, $E_{c}^{-}$). The absorption spectrum of Si is also shown in Figure 3d.

The absorption spectrum (*α*) is an essential factor for the PCE analysis of a solar cell. In this study, the absorption spectrum of the bulk GaNAsP was calculated based on the joint density of states derived from the band anti-crossing model. The calculated three-section absorption spectra of GaP-lattice-matched GaNAsP are plotted in Figure 3. The absorption spectrum of valence band to $E_{c}^{+}$ band transition, *α* ($E_{v}$, $E_{c}^{+}$) is similar to the absorption spectrum of another III-V compounds of valence band to CB transition from parabolic approximation. According to the GaNAsP band structure calculated by Kudrawiec et al. [21], the distance between the $E_{c}^{+}$ state and the N level greatly increases at a large *k* value. Hence, the dispersion relation of the $E_{c}^{+}$ state is similar to the parabolic approximation dispersion relation, with a greater effective mass. However, the $E_{c}^{-}$ state approaches the N level with an increasing value of *k*. This effect results in quick optical absorption saturation and then decay corresponding to a shorter wavelength. Therefore, the absorption spectrum of valence band to $E_{c}^{-}$ band transition, *α* (*E*_v_, $E_{c}^{-}$), is quite different from the other III-V interband absorption spectrum. Noticeably, if the parabolic approximation is used with Δ*E* ($E_{c}^{-}$, *E*_v_) as the bandgap for GaNAsP absorption spectrum calculation, the error increases with increasing N concentration. Because of the dispersion relation property of the $E_{c}^{-}$ state and Duboz’s modification, the calculated absorption spectrum for $E_{c}^{-}$ band to $E_{c}^{+}$ band transition, *α* ($E_{c}^{-}$, $E_{c}^{+}$), is an intraband transition. The strength of *α* ($E_{c}^{-}$, $E_{c}^{+}$) is relatively low compared with that of *α* ($E_{v}$, $E_{c}^{-}$).

If the $E_{c}^{-}$ band is treated as an IB, the corresponding three-sections absorption spectra for N = 0.01 to N = 0.1 are as shown in Figure 3a–c. The absorption strength of *α* ($E_{c}^{-}$, $E_{c}^{+}$) is weaker than *α* ($E_{v}$, $E_{c}^{-}$) by at least one order of magnitude. According to the theory of an IBSC, this will result in a weak current contribution from the two sub-bandgaps. The major contribution is from the VB to the $E_{c}^{-}$ band absorption. The PCE with the $E_{c}^{-}$ band as the IB was analyzed using the method and PIN structure proposed by Yoshida et al. [28]. The structure had 0.5 μm thick GaP as the P region, 1 μm thick GaNAsP as the I-region, and 2 μm thick GaP as the N-region. The doping concentrations of P- and N- regions were 7 × 10^17^ cm^−3^ and 6 × 10^17^ cm^−3^, respectively. The doping concentration of the I-region depends on the absorption strength of the transition from the VB to the IB and from the IB to the CB. The calculated PCE of the proposed GaNAsP IBSC was approximately 11.4% for different mole fractions of nitrogen. The open voltage is almost the same because GaP is used in each cell’s P- and N- regions. The variation in short current density for GaNAsP-based IBSC with different N concentrations has little difference of value. Consequently, the variation in PCE at different N concentrations of bulk-GaNAsP-based IBSC is less than 0.02%. Moreover, after replacing the I region with GaP, the calculated PCE of the GaP solar cell is 10.6%, with the same P and N region doping concentrations and the same geometric parameters. The conversion efficiency gain is only 0.8%. This verifies that the two-section absorption with regard to the IB is not matched. Therefore, the extra PCE gain resulting from the $E_{c}^{-}$ band as the IB is unremarkable.

If GaNAsP is treated as a single gap material with a smaller bandgap, Δ*E* ($E_{c}^{-}$, *E*_v_), the absorption spectrum should be obtained by summing *α* ($E_{v}$, $E_{c}^{+}$) and *α* ($E_{v}$, $E_{c}^{-}$), as plotted in Figure 3d. The separation between the $E_{c}^{-}$ band and the $E_{c}^{+}$ band becomes larger with an increase in the mole fraction of N. This agrees with the dispersion relation derived from the band anti-crossing model given in Equation (3). The variation in Δ*E* ($E_{c}^{-}$, *E*_v_) with N mole fraction is plotted in Figure 2b. The bandgap narrowing increases the short current of the solar cell but decreases the open voltage of a GaNAsP-based solar cell.

### 3.2. GaNAsP/Si Tandem Cell

**Figure 4:**
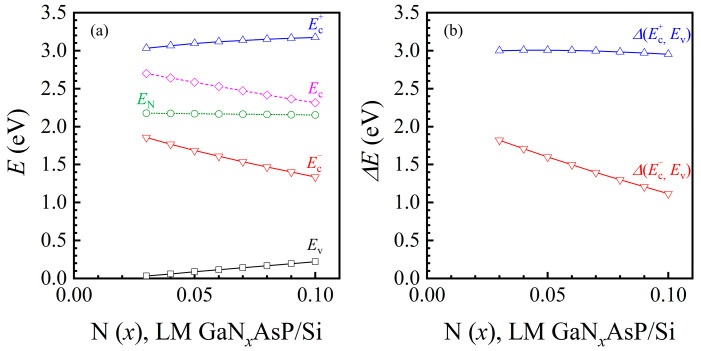
(**a**) Calculated $E_{c}^{+}$ and $E_{c}^{-}$ band edges of Si-lattice-matched GaN*_x_*AsP*_y_* (LM GaN*_x_*AsP/Si), as a function of N mole fraction. The reference energy level is the valence band edge of GaP. The valence band edge ($E_{v}$), the conduction band edge ($E_{c}$), and the nitrogen level ($E_{N}$) of the original GaAsP*_y_*; (**b**) Calculated Si-lattice-matched GaN*_x_*AsP energy gap (Δ*E*) between $E_{c}^{+}$ and valence band (*E*_v_), Δ*E* ($E_{c}^{+}$, $E_{v}$), and between $E_{c}^{-}$ and $E_{v}$, Δ*E* ($E_{c}^{-}$, $E_{v}$), as a function of N mole fraction.

In this section, we analyze the concentrations of GaNAsP with a lattice constant equal to that of Si, considering N mole fraction varying from 0.03 to 0.1. A lattice-matched GaNAsP/Si tandem cell was designed. Some assumptions are applied to analyze the PCE of the tandem cell herein: the first, there is no inter-layer between the subcells; the second, the bottom cell acts as the top cell except for the modified generation term; and the third, the short current density of the tandem cell depends on the smaller subcell. The tandem cell’s open voltage is the sum of the subcells’ open voltages. The filling factor is assumed to be 1, i.e., the PCE of the tandem cell is the product of short current density and open voltage over the total incident power. The designed structure contains GaNAsP (PIN junction) and Si (PN junction). The P-region, I-region, and N-region of GaNAsP, were 0.5 μm, 1 μm, and 2 μm thick. The doping concentrations of P- and N- regions of GaNAsP cell were 7 × 10^17^ cm^−3^ and 6 × 10^17^ cm^−3^, respectively, with a undoped I region. The P- and N- regions of Si had the same thickness. The doping concentrations of P- and N- regions of Si cell were 7 × 10^17^ cm^−3^ and 6 × 10^17^ cm^−3^, respectively. The thickness of the GaNAsP top cell was fixed as 3.5 μm, and the Si bottom cell thickness was varied from 1 μm to 8 μm. As the P- and N-region materials are GaNAsP, the stimulated electrons in the $E_{c}^{-}$ band from the VB can contribute to the current density without absorbing secondary light to the $E_{c}^{+}$ band to flow into the external circuit. Therefore, the effective absorption spectrum is the sum of *α* ($E_{v}$, $E_{c}^{+}$) and *α* ($E_{v}$, $E_{c}^{-}$). The calculated absorption spectra (*α*) for bulk Si-lattice-matched GaNAsP are similar to the spectrum of the bulk GaP-lattice-matched GaNAsP, as depicted in Figure 3. Moreover, the phosphorus concentration of Si-lattice-matched GaNAsP is higher than that of GaP-lattice-matched GaNAsP, as shown in Figure 1. Therefore, the Si-lattice-matched GaNAsP has higher Δ*E* ($E_{c}^{+}$, $E_{v}$) and lower Δ*E* ($E_{c}^{-}$, $E_{v}$), with minor variations. (53 meV for Δ*E* ($E_{c}^{+}$, $E_{v}$) and 705 meV for Δ*E* ($E_{c}^{-}$, *E*_v_), as shown in Figure 4.

**Figure 5:**
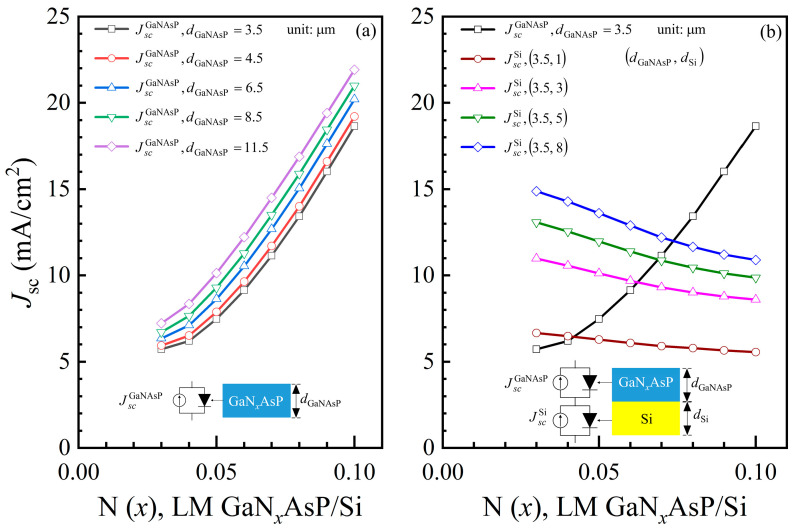
(**a**) Calculated short current densities of Si-lattice-matched GaN*_x_*AsP (LM GaN*_x_*AsP/Si) cells short current densities ($J_{sc}^{\mathrm{GaNAsP}}$) as a function of N mole fraction; (**b**) Short current densities (*J*_*sc*_) of 3.5 μm thick GaNAsP top cell ($J_{sc}^{\mathrm{GaNAsP}}$) and Si bottom cell ($J_{sc}^{\mathrm{Si}}$) with different thicknesses as a function of N concentration.

Figure 5a shows the short current density of Si-lattice-matched GaN*_x_*AsP ($J_{sc}^{\mathrm{GaNAsP}}$) with different thicknesses as a function of N mole fraction. The short current density increases with the increase in thickness and N mole fraction. A thicker cell has a longer travel distance for photon absorption. Meanwhile, higher N mole fraction GaNAsP has a smaller bandgap, Δ*E* ($E_{c}^{-}$, *E*_v_), for a broader absorption spectrum. These two effects result in a higher short current density. Figure 5b shows the short current density of each subcell in GaNAsP/Si tandem cell with 3.5 μm thick GaNAsP, $J_{sc}^{\mathrm{GaNAsP}}$ for the GaNAsP top cell, and $J_{sc}^{\mathrm{Si}}$ for the Si bottom cell. For the Si cell, the short current density increases with thicker cells because the thickness is not sufficient for full absorption. The slight drop in the Si short current density occurs because Si is the bottom cell and the absorption spectrum of Si overlaps with *α* of GaNAsP, as shown in Figure 3d. Therefore, the Si bottom cell solely utilizes the remaining photon after GaNAsP absorption in the overlapping range of the spectrum. The lower of the two short current density values of the GaNAsP cell and the Si cell is set as the tandem cell short current density for the current-matching condition. The open voltage is the sum of the voltages of the GaNAsP cell and the Si cell.

**Figure 6:**
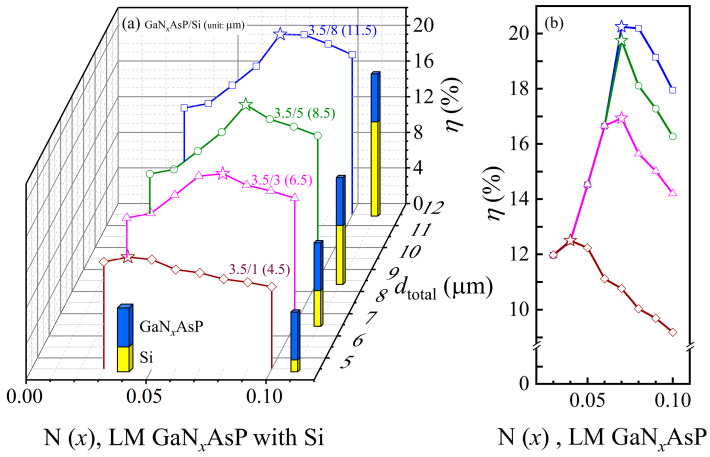
(**a**) PCEs (*η*) of the lattice-matched GaN*_x_*AsP/Si tandem cell as a function of N concentration (*x*) with the different total thickness (*d*_total_). The schematics of the tandem cell are plotted in the figure. The thickness of each cell is labeled above the curve; (**b**) PCEs of GaNAsP/Si tandem cell as a function of nitrogen concentration (*x*).

The PCEs of the tandem cell as a function of N concentration with the different total thickness (*d*_total_) are plotted in Figure 6a. The schematics of the proposed Si-lattice-matched GaNAsP/Si tandem cell are shown in the figure. The blue pillar represents the GaNAsP top cell, and the yellow pillar represents the Si bottom cell. The figure shows that the maximum PCE of Si-lattice-matched GaNAsP/Si tandem will be at different N concentrations with cascading different thicknesses of the Si bottom cell. Figure 6b shows the PCEs of the tandem cell as a function of N(*x*) in a 2D diagram. Because the short current density of the tandem cell is limited to the lower one of each cell, the maximum PCE is achieved when the short current density of the GaNAsP cell and the Si cell are equal. Because of the sampling rate limitation, the nitrogen concentration of tandem cells with maximum PCE is closest to the current-matched concentration, as shown in Figure 5b. The Si bottom cell only utilizes the remaining photon after GaNAsP absorption in the overlapping range of the spectrum. However, with the Si thickness increasing, the increase in PCE reveals that the Si bottom is not thick enough for full absorption. Thicker Si bottom cell can generate a higher short current density to be current-matched with GaNAsP with higher N concentration.

**Figure 7:**
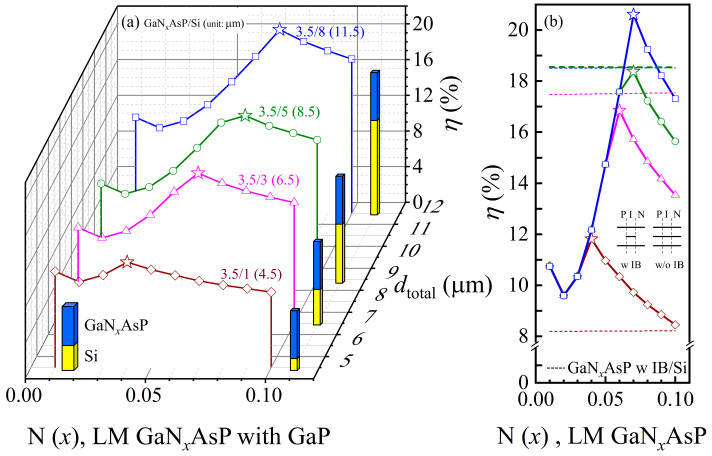
(**a**) PCEs (*η*) of the GaP-lattice-matched GaN*_x_*AsP/Si tandem cell as a function of N concentration (*x*) with the different total thickness (*d*_total_). The schematic diagrams of the tandem cell are plotted in the figure. The thickness of each cell is labeled above the curve; (**b**) PCEs of GaP-lattice-matched GaNAsP/Si tandem cell as a function of nitrogen concentration (*x*). The dashed lines represent the PCE of GaP-lattice-matched GaN*_x_*AsP IBSC with $E_{c}^{-}$ band as IB and GaP as the top cell’s P- and N-region’s material. The schematic diagrams of band structure with or without IB are also plotted.

The difference between the lattice constants of GaP and the lattice constant of Si is small (0.02Å). In this section, the GaP-lattice-matched GaNAsP was also considered as the top cell. To utilize the GaNAsP-based IBSC, the materials of the P- and N- regions were set as GaP. The PCEs of the tandem cell as a function of N concentration with the different total thickness (*d*_total_) are plotted in Figure 7a. The schematic diagrams of the proposed GaP-lattice-matched GaNAsP/Si tandem cells are plotted in the figure. The blue pillar represents the GaNAsP top cell, and the yellow pillar represents the Si bottom cell. The figure shows that the maximum PCE of GaP-lattice-matched GaNAsP/Si tandem will have different nitrogen concentrations for different cascading thicknesses of the Si bottom cell. Because the smaller short current density of each cell defines the short current density of the tandem cell, the maximum PCE is achieved when the GaNAsP cell and the Si cell have the same short current density. Because of the sampling rate limitation, the N concentration of tandem cells with maximum PCE is the concentration closest to the current-matched concentration. In Figure 7b, the dashed lines represent those PCEs of GaP-lattice-matched GaNAsP-based IBSC/Si tandem cells in different total thickness (*d*_total_), which color represents for the corresponding same *d*_total_ as indicated in Figure 7a. It shows that the PCE cannot be enhanced as N concentration increased. As shown in Figure 7b, the PCE of tandem cells with different Si bottom cell thicknesses (1–5 μm) increases, which means that the thickness is still not sufficient for complete absorption. When the thickness of Si is over 5 μm, the PCE of the tandem cell clamps because the short current density of Si bottom cell exceeds the short current density of the top cell, consisting of the GaNAsP-based IBSC. However, the clamping phenomenon does not happen for GaNAsP/Si tandem cell for which the materials of the P- and N- regions are GaNAsP in the top cell. This effect means the short current density of the tandem cell is limited to the Si bottom cell. The relatively high PCE of GaP-lattice-matched GaNAsP-based IBSC/Si is from the higher open voltage from the IBSC, except for the tandem cell with 8 μm thickness Si which provides higher short current density.

**Figure 8:**
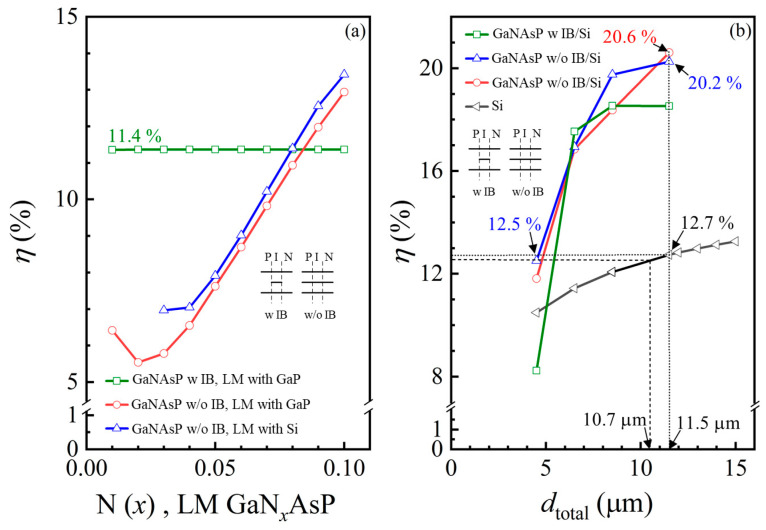
(**a**) PCEs (*η*) of the GaP-lattice-matched GaN*_x_*AsP-based IBSC (GaNAsP w IB, LM with GaP), the GaP-lattice-matched GaN*_x_*AsP (GaNAsP w/o IB, LM with GaP), and the Si-lattice-matched GaN*_x_*AsP (GaNAsP w/o IB, LM with Si) as a function of N(*x*) with the different total thickness (*d*_total_); (**b**) The comparison between PCEs of GaNAsP/Si tandem cell and Si cell at different total thickness (*d*_total_). Each point of the GaNAsP/Si tandem cell represent the maximum PCE of each thickness combination.

In Figure 8a, the PCE of the GaP-lattice-matched GaNAsP top cell was firstly analyzed. Because of the large strength difference between *α* ($E_{c}^{-}$, $E_{c}^{+}$) and *α* ($E_{v}$, $E_{c}^{-}$), the major contribution to the current is from *α* (*E*_v_, $E_{c}^{+}$). However, the variation in Δ*E* ($E_{c}^{+}$, $E_{v}$) with the mole fraction of nitrogen is relatively slight (61 meV), as shown in Figure 2b. Therefore, the PCE of GaNAsP-based IBSC with GaP/GaNAsP/GaP PIN structure is 11.4% with different mole fractions of nitrogen. When the materials of P- and N- regions are substituted by GaNAsP, the GaNAsP can be treated as a single bandgap (Δ*E* ($E_{c}^{-}$, *E*_v_)) material, with the absorption spectrum as shown in Figure 3d. Therefore, as indicated by green line with symbol of (□), the PCE of GaN*_x_*AsP-based IBSC (GaNAsP w IB, LM with GaP) is almost invariant and kept at *η* = 11.4% with different N concentrations. The red line with symbol of (○) shows that the PCE of GaN*_x_*AsP cell (GaNAsP w/o IB, LM with GaP) increases with the increase in nitrogen concentration, except for GaN_0.01_AsP. From Figure 2b and Figure 3, the strength of *α* ($E_{v}$, $E_{c}^{+}$) and Δ*E* ($E_{c}^{-}$, *E*_v_) decreases with the increase in N concentration, whereas the strength of *α* ($E_{v}$, $E_{c}^{-}$) increases with the increase in N concentration. The GaN_0.01_AsP-based cell has a larger bandgap with a larger open voltage. This effect causes a GaN_0.01_AsP-based cell to have a higher PCE than a GaN_0.03_AsP-based cell. The blue line with symbol of (△) shows that the PCE of Si-lattice-matched GaN*_x_*AsP cell (GaNAsP w/o IB, LM with Si) as a function of N concentration. The PCE grows with the increase in N concentration due to the shrinking of bandgap, Δ*E* ($E_{c}^{-}$, *E*_v_).

Figure 8b depicts the maximum PCE of different thickness combinations GaNAsP/Si tandem cell as a function of the cell thickness. The green line represents the GaNAsP-based IBSC as the top cell of the tandem cell with GaP as the material of P- and N- regions (GaNAsP w IB/Si). The red and blue lines represent GaNAsP/Si tandem cells, with GaNAsP as the material of P- and N- regions of the top cells. The red line represents the GaP-lattice-matched GaNAsP/Si tandem cell, and the blue line represents the Si-lattice-matched GaNAsP/Si tandem cell. The PCE of the Si cell as a function of cell thickness is also plotted as the black line with the triangle symbols in Figure 8b. Among the other tandem cells, 3.5 μm Si-lattice-matched GaN_0.04_AsP/1 μm Si tandem cell has the same PCE, 12.5%, as a 10.5 μm thick Si solar cell, solely having a total thickness of 5.5 μm, depicted as a dashed line in Figure 8b. Moreover, the maximum efficiency of 20.6% was obtained with a 3.5 μm GaP-lattice-matched GaN_0.07_AsP/8 μm Si tandem cell, with a 11.5 μm total thickness, whereas a 11.5 μm thick Si cell has 12.7% PCE, depicted as dotted-line in Figure 8b. A 3.5 μm Si-lattice-matched GaN_0.07_AsP/8 μm Si tandem cell has 20.2% PCE, with 11.5 μm total thickness. Thus, the GaNAsP/Si tandem cells are suitable for ultra-thin film flexible solar cells design, with higher PCE and less thickness than Si cells.

**Figure 9:**
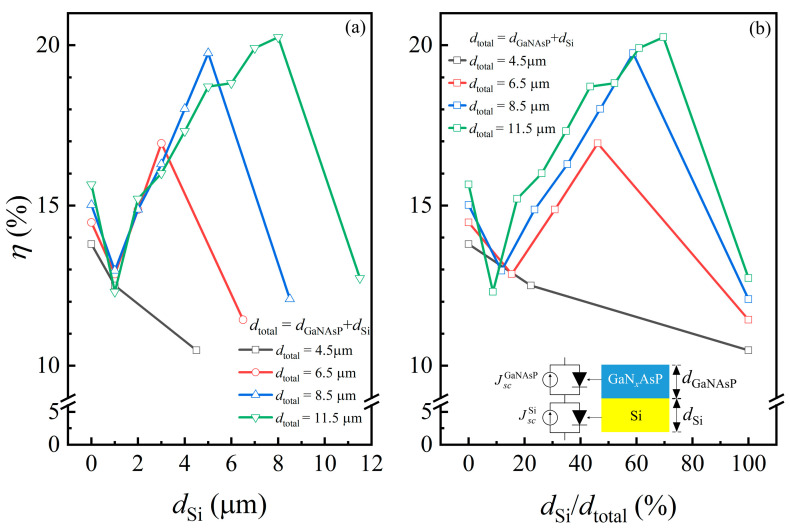
Maximum PCEs (*η*) of the Si-lattice-matched GaN*_x_*AsP/Si tandem cell with a different total thickness (*d*_total_) as a function of (**a**) the thickness of the Si bottom cell; (**b**) the thickness ratio of the Si bottom cell. The schematic structure diagram and circuit diagram of the GaN*_x_*AsP/Si tandem cell are also shown in the figure.

Figure 9 shows the maximum PCE of different thickness combinations of GaN_x_AsP/Si as a function of (a) thickness and (b) thickness ratio of the Si bottom cell. The starting point represents the PCE of the GaNAsP cell, and the end point represents the PCE of the Si cell of each line. Except for the 4.5 μm cell, it shows that the other GaNAsP/Si tandem cells have higher PCE than the GaNAsP cell and Si cell. For thicker cells, the thickness ratio of the Si bottom cell should be higher for a higher tandem cell PCE. The suggested thickness ratio of the Si bottom cell is between 40% to 70% for the sub-12 μm GaNAsP/Si tandem cell to achieve maximum PCE.

## 4. Conclusions

This study analyzed the PCE of a GaNAsP cell. We calculated the three-section absorption spectrum from the formula derived from the band anti-crossing model. The results showed a significant strength difference between *α* ($E_{v}$, $E_{c}^{-}$) and *α* ($E_{c}^{-}$, $E_{c}^{+}$). Therefore, the GaNAsP-based IBSC had no significant PCE enhancement compared with the GaP cell. However, we can utilize the GaNAsP as a single-bandgap material in PIN structure solar cells by selecting the P- and N-region materials. GaNAsP can be lattice-matched with GaP or Si. This study used this property to design a lattice-matched GaNAsP/Si tandem cell. We analyzed the mole fraction variation and different subcell thickness combinations in GaNAsP/Si tandem cells. The results showed that the lattice-matched GaNAsP/Si tandem cell had a higher PCE than that of a Si cell. An 11.5 μm tandem cell had a 7.5% PCE enhancement over that of a Si cell with the same thickness. Meanwhile, the 4.5 μm GaNAsP/Si tandem cell had the same PCE as a 10.7 μm Si cell, and the tandem cell had a 6.2 μm thickness reduction. With the superiority, we think that the lattice-matched GaNAsP/Si tandem cell is suitable for ultrathin Si-based solar cells. Meanwhile, the thickness ratio of the tandem cell’s Si subcell was analyzed. The sub-12 μm tandem cell would have a maximum PCE with a ratio of 40% to 70% of the Si cell. The exact ratio depended on the tandem cell thickness. The thicker tandem cell needed a higher thickness ratio of *d*_Si_/*d*_total_ to achieve the maximum PCE.

It is known that growth of high crystal quality GaNAsP alloy consisting in high N concentration is difficult due to the different properties between N and P (As), such as the atomic size and the electronegativity. We report in the case of a 3.5 mm GaNAsP/8 μm Si tandem cell, it requires an N concentration of 7% for reaching the PCE to 20.2%. Although, there is no report for growing GaNAsP alloy with a such high N concentration. Eventually, the idea presented in this study can benefit in evaluating the possibility of using dilute-nitride GaNAsP to make the Si-based solar cell keeping in an ultrathin thickness and performing a much higher PCE as well.

## Data Availability

Data underlying the results presented in this paper are not publicly available at this time but may be obtained from the authors upon reasonable request.

## References

[1] Priolo F., Gregorkiewicz T., Galli M., Krauss T.F. (2014). Silicon Nanostructure for Photonics and Photovoltaics. Nat. Nanotechnol..

[2] Petermann J.H., Zielke D., Schmidt J., Haase F., Rojas E.G., Bendel R. (2012). 19%-Efficient and 43 mm-Thickness Crystalline Si Solar Cell Form Layer Transfer Using Porous Silicon. Prog. Photov. Res. Appl..

[3] Wang S., Weil B.D., Li Y., Wang K.X., Garnett E., Fan S., Cui Y. (2013). Large-Area Free-Standing Ultrathin Single-Crystal Silicon as Processable Materials. Nano Lett..

[4] Cruz-Campa J.L., Nielson G.N., Resnick P.J., Sanchez C.A., Clews P.J., Okandan M., Friedmann T., Gupta V.P. (2011). Ultrathin Flexible Crystalline Silicon: Microsystems-Enabled Photovoltaics. IEEE J. Photovolt..

[5] Pudasaini P.R., Sharma M., Ruiz-Zepeda F., Ayon A.A. Proceedings of the Name of Ultrathin, Flexible, Hybrid Solar Cells in Sub-Ten Micrometers Single Crystal Silicon Membrane. Proceedings of the IEEE 40th Photovoltaic Specialist Conference (PVSC).

[6] Tan X.Y., Sun L., Zhang G.R., Deng C., Tu Y.T., Guan L. (2019). Absorption Enhancement of Ultrathin Crystalline Silicon Solar Cells with Dielectric Si_3_N_4_ Nanostructures. Commun. Theor. Phys..

[7] Wang C., Zhao S., Bian F., Du D., Wang C., Xu Z. (2020). Absorption Enhancement of Ultrathin Crystalline Silicon Solar Cells with Frequency Upconversion Nanosphere Arrays. Commun. Theor. Phys..

[8] Tang Q., Shen H., Yao H., Gao K., Jiang Y., Liu Y.L.Y., Zhang L., Ni Z., Wei Q. (2019). Superiority of Random Inverted Nanopyramid as Efficient Light Trapping Structure in Ultrathin Flexible C-Si Solar Cell. Renew. Energy.

[9] Li Y., Li Y., Wang X., Wang Y. (2019). Ultrathin C-Si Solar Cells Based on Microcavity Light Trapping Scheme. Opt. Quant. Electron..

[10] Hadibrata W., Es F., Yerci S., Turan R. (2018). Ultrathin Si Solar Cell with Nanostructured Light Trapping by Metal Assisted Etching. Sol. Energy Mater Sol. Cells.

[11] Jangioy A., Bahador H., Heidarzadeh H. (2019). Design of an Ultra-Thin Silicon Solar Cell Using Localized Surface Plasmonic Effects of Embedded Paired Nanoparticles. Opt. Commun..

[12] Sobhani F., Heidarzadeh H., Bahador H. (2020). Efficiency Enhancement of an Ultra-Thin Film Silicon Solar Cell Using Conical-Shaped Nanoparticles: Similar to Superposition (top, middle, and bottom). Opt. Quant. Electron..

[13] Köhnen E., Jošt M., Morales-Viches A.B., Tockhom P., Al-Ashouri A., Macco B., Kegelmann L., Korte L., Rech B., Schlatmann R. (2019). Highly Efficient Monolithic Perovskite Silicon Tandem Solar Cells: Analyzing the Influence of Current Mismatch on Device Performance. Sustain. Energy Fuels.

[14] Subbiah A.S., Isikor F.H., Howells C.T., Bastiani M.D., Liu J., Aydin E., Furlan F., Allen T.G., Xu F., Zhumagali S. (2020). High-Performance Perovskite Single-Junction and Textured Perovskite/Silicon Tandem Solar Cells Via Slot-Die-Coating. ACS Energy Lett..

[15] Nacer S., Aissat A. (2016). Simulation and Optimization of Current and Lattice Matching Double-Junction GaNAsP/Si Solar Cells. Superlattic Microstruct..

[16] Zhang X., Lin K., Xie H., Wang Y. (2020). Theoretical Study on Potential Performance of Lattice-Matched Monolithic GaNP/GaNAsP/Si Triple-Junction Solar Cell. J. Phys. D Appl. Phys..

[17] Sertel T., Ozen Y., Cetin S.S., Ozturk M.K., Ozcelik S. (2018). Structure, Optical and Electrical Characterization of Dilute Nitride GaP_1-*x*-*y*_As*_y_*N*_x_* Structures Grown on Si and GaP Substrates. J. Mater. Sci. Mater Electron..

[18] Yu K.M., Walukiewicz W., Ager J.W., Bour D., Farshchi R., Dubon O.D., Li S.X., Sharp I.D., Halle E.E. (2006). Multiband GaNAsP Quaternary Alloys. Appl. Phys. Lett..

[19] Zelazna K., Gladysiewicz M., Polak M.P., Létoublon S.A.A., Cornet C., Durand O., Walukiewicz W., Kudrawiec R. (2017). Nitrogen-Related Intermediate Band in P-Rich GaN*_x_*P*_y_*As_1-*x*-*y*_ Alloys. Sci. Rep..

[20] Luque A., Marti A. (1997). Increasing the Efficiency of Ideal Solar Cells by Photon Induced Transitions at Intermediate Levels. Phys. Rev. Lett..

[21] Kudrawiec R., Luce A.V., Gladysiewicz M., Ting M., Kuang Y.J., Tu C.W., Dubon O.D., Yu K.M., Walukiewicz W. (2014). Electronic Band Structure of GaN*_x_*P*_y_*As_1−*x*−*y*_ Highly Mismatched Alloys: Suitability for Intermediate-Band Solar Cells. Phys. Rev. Appl..

[22] Jussila H., Kivisaari P., Lemettinen J., Tanaka T., Sopanen M. (2015). Two-Photon Absorption in GaAs_1−*x*−*y*_P*_y_*N*_x_* Intermediate-Band Solar Cells. Phys. Rev. Appl..

[23] Wu J., Shand W., Walukiewicz W. (2002). Band Anticrossing in Highly Mismatched III-V Semiconductor Alloys. Semicond. Sci. Technol..

[24] Vurgaftman I., Meyer J.R. (2001). Band Parameters for III-V Compound Semiconductors and Their Alloys. J. Appl. Phys..

[25] Vurgaftman I., Meyer J.R. (2003). Band Parameters for Nitrogen-Containing Semiconductors. J. Appl. Phys..

[26] Chung S.L. (2009). Physics of Photonic Devices.

[27] Duboz J.Y. (2007). Energy Levels and Intersubband Transition in InGaAsN/AlGaAs Quantum Wells. Phys. Rev. B.

[28] Yoshida K., Okada Y., Sano N. (2012). Device Simulation of Intermediate Band Solar Cells: Effects of Doping and Concentration. J. Appl. Phys..

